# Remarkable electronic and optical anisotropy of layered 1T’-WTe_2_ 2D materials

**DOI:** 10.3762/bjnano.10.170

**Published:** 2019-08-20

**Authors:** Qiankun Zhang, Rongjie Zhang, Jiancui Chen, Wanfu Shen, Chunhua An, Xiaodong Hu, Mingli Dong, Jing Liu, Lianqing Zhu

**Affiliations:** 1 School of Instrument Science and Opto-Electronics Engineering, Beijing Information Science and Technology University, No.12 Xiaoying East Road, Beijing, China; 2 School of Precision Instrument and Opto-Electronics Engineering, Tianjin University, 92 Weijin road, Tianjin 300072, China

**Keywords:** 1T’-WTe_2_, 2D material, anisotropic, light polarization, optoelectrical

## Abstract

Anisotropic 2D materials exhibit novel optical, electrical and thermoelectric properties that open possibilities for a great variety of angle-dependent devices. Recently, quantitative research on 1T’-WTe_2_ has been reported, revealing its fascinating physical properties such as non-saturating magnetoresistance, highly anisotropic crystalline structure and anisotropic optical/electrical response. Especially for its anisotropic properties, surging research interest devoted solely to understanding its structural and optical properties has been undertaken. Here we report quantitative, comprehensive work on the highly anisotropic, optical, electrical and optoelectronic properties of few-layer 1T’-WTe_2_ by azimuth-dependent reflectance difference microscopy, DC conductance measurements, as well as polarization-resolved and wavelength-dependent optoelectrical measurements. The electrical conductance anisotropic ratio is found to ≈10^3^ for a thin 1T’-WTe_2_ film, while the optoelectronic anisotropic ratio is around 300 for this material. The polarization dependence of the photo-response is ascribed to the unique anisotropic in-plane crystal structure, consistent with the optical absorption anisotropy results. In general, 1T’-WTe_2_, with its highly anisotropic electrical and photoresponsivity reported here, demonstrates a route to exploit the intrinsic anisotropy of 2D materials and the possibility to open up new ways for applications of 2D materials for light polarization detection.

## Introduction

The first exfoliation of graphene [1] attracted extensive interest in 2D materials such as black phosphorus (BP) [2–3], hexagonal boron nitride (h-BN) [4–5] and transition metal dichalcogenides (TMDCs) with a common chemical formula MX_2_ [6–10]. Due to the many excellent electronic, mechanical and optoelectronic properties, TMDCs are highly attractive for fundamental studies of novel physical phenomena and for applications ranging from electronics [11] and photonics [12–13] to sensing [14–15] and actuation [16] at the nanoscale.

Recently, tungsten telluride (WTe_2_), as a member of the TMDC family, has drawn tremendous research interest since the discovery of its large non-saturating positive magnetoresistance (MR) behavior, which was attributed to its perfect electron–hole compensation. Theoretical calculations and experiments have indicated that WTe_2_ has a complicated electronic structure with more than two bands presenting at the Fermi level [17]. Meanwhile, it was believed to a wonderful candidate for the type-II Weyl semimetal [18] and quantum spin Hall insulator material [19]. Additionally, many other remarkable properties of WTe_2_ have also been revealed, such as the temperature-driven Lifshitz transition [20], pressure-induced superconductivity [21], linear Nernst response [22], etc. Interestingly, although all of the TMDCs have the same formula, the atomic structure of 1T’-phase WTe_2_ is totally different from the other TMDCs. 1T’-WTe_2_ exhibits a distorted structure relative to the 1T’ phase. Both Raman [23–24] and first-principles [25–26] calculations have been used to indicate that monolayer 1T’-WTe_2_ has a highly in-plane anisotropic crystal structure, in addition to anisotropic electrical, thermal and optical properties [23–26]. However, most of the results are based on theoretical demonstrations of the anisotropic phenomenon, rather than further quantitative data for the characterization the natural anisotropy of 1T’-WTe_2_.

In this paper, we present a combined experimental and quantitative study on the anisotropic optical and electronic properties of mechanically isolated 1T’-WTe_2_. Through a systematic characterization including Raman spectroscopy, X-ray photoelectron spectroscopy and azimuth-dependent reflectance difference microscopy (ADRDM), we firstly identified the 1T’-phase WTe_2_ to have an optical anisotropic crystal structure. Secondly, a 12-electrode-structure was designed for the evaluation the electrical anisotropy of 1T’-WTe_2_, and the results demonstrated up to 10^3^ times relative electrical anisotropy. Subsequently, the photo-electrical conductance was studied to further explore the anisotropic photo-electric property. Our results revealed an anisotropic photo-electrical conductance with a maximum ratio of 4.33 among the 12 directions. Finally, a photodetector with twelve electrodes was used to distinguish the different wavelengths in the visible spectrum, demonstrating that 1T’-WTe_2_ shows promising application as an anisotropic photodetector.

## Results and Discussion

### Characterization of 1T’- WTe_2_

**Figure 1:**
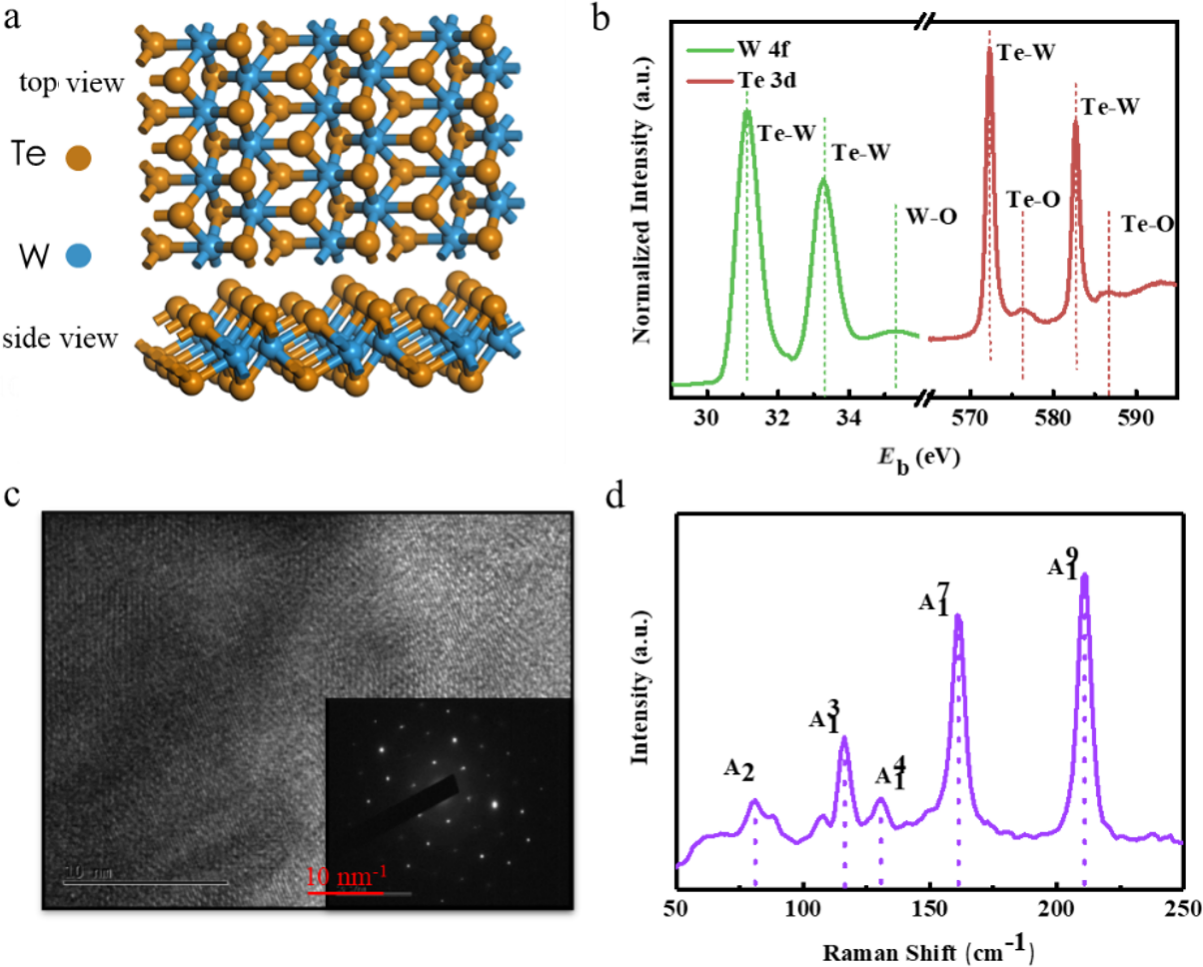
Topological phase and characterization of 1T’-WTe_2_. a) Side and top views of lattice structures of 1T’-WTe_2_. Brown spheres: tellurium atoms, blue spheres: tungsten atoms. b) XPS spectrum of W 4f (green line) and Te 3d (red line) peaks. c) TEM image and SEAD pattern of WTe_2_, revealing the crystal structure of the 1T’ phase, with lattice parameters *a* ≈ 3.49 Å and *b* ≈ 6.32 Å, respectively. d) Raman spectrum of a 1T’-WTe_2_ flake.

Figure 1a shows the typical orthorhombic crystalline structure of 1T’-WTe_2_. The tungsten atoms deviate from the ideal octahedral sites in the octahedron due to the very strong chemical bonding, forming the distorted octahedral structure. Consequently, the Te atom layers become buckled in the distorted octahedral structure.

X-ray photoelectron spectroscopy (XPS) was employed to verify the elemental distribution and the bonding types of the exfoliated 1T’-WTe_2_ nanoflakes. The prominent W 4f peaks were observed at 31.3 eV and 33.5 eV, corresponding to 4f_7/2_, and 4f_5/2_, respectively, of the W–Te bonds (Figure 1b left in green), while the Te 3d spectrum has two peaks at 572.7 eV (3d_5/2_) and 582.9 eV (3d_3/2_), respectively, corresponding to W–Te bonds (Figure 1b right in red) [27]. The crystalline parameters of WTe_2_ were further studied through transmission electron microscopy (TEM), where a typical high-resolution TEM image displayed in Figure 1c shows the distorted 1T’ atomic phase, and the selected area electron diffraction (SEAD) pattern illustrated in Figure 1c, which demonstrates the rectangular symmetry of 1T’-WTe_2_ with space group Pmn2_1_. Furthermore, graphene diffraction spots were utilized for calibrating the SAED pattern, and we measured the lattice parameters to be *a* ≈ 3.49 Å and *b* ≈ 6.32 Å, respectively, which are in excellent agreement with a previous report [28]. In order to gain further information on the crystal structure, Raman spectroscopy was performed on 1T’-WTe_2_ nanosheets, as shown Figure 1d. All of the peak positions are consistent with those reported from exfoliated thin nanosheet samples previously reported [29–30]. Additionally, a similar negative-direction shift of the peak positions is witnessed as the sample thickness increases [29].

### Anisotropic crystal structure identification by azimuth-dependent reflectance difference microscopy (ADRDM)

The optical anisotropy of 1T’-WTe_2_ was characterized by ADRDM. The ADRDM measurements were conducted by measuring the normalized reflectance difference of two arbitrary orthogonal directions at normal incidence, which can provide a direct, accurate visualization of the anisotropy of low-symmetry 2D materials. In detail, when linearly polarized incident light impinges onto the 1T’-WTe_2_ crystal, as expected, an obvious phase retardance (Δδ) and reflectance difference (Δ*R*) appears between the two optical axes. The significant reflectance difference along the *x*- (*R_x_*) and *y*- *(R_y_)* axis is defined by the following formula [30]:

[1]
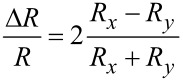


where *x* and *y* are the reference orientations of the ADRDM measurement setup. When we change the relative direction θ (incidence angle) between the ADRDM and the orthorhombic crystal, this results in a dimensionless value *N*(θ), which periodically varies with θ in the following way:

[2]
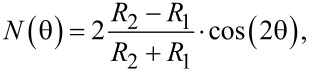


where *R*_2_ and *R*_1_ are the reflectance coefficients associated with the natural orthorhombic crystal.

**Figure 2:**
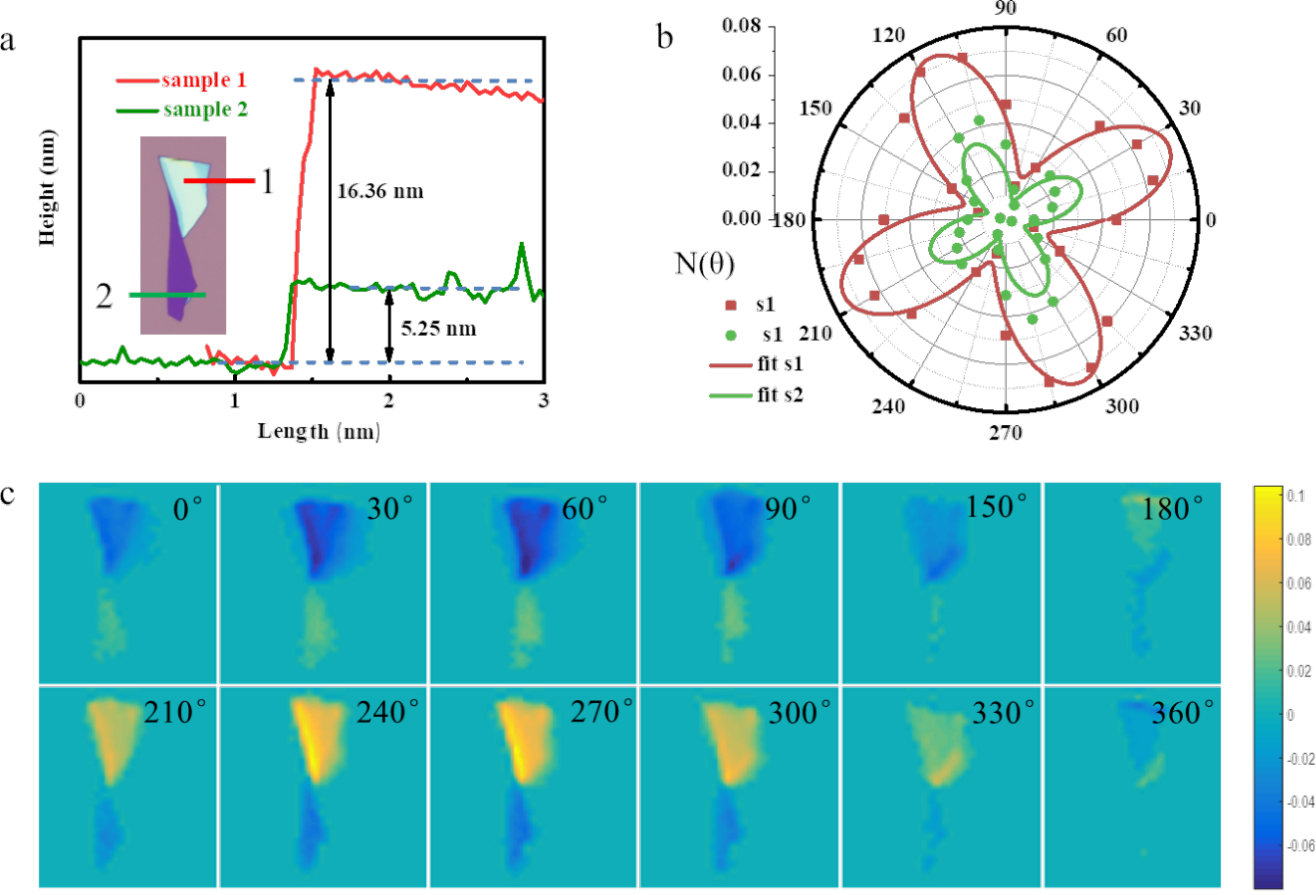
Identification of the lattice anisotropy of an exfoliated 1T’-WTe_2_ flake. a) AFM image of the 1T’-WTe_2_ flake transferred onto a Si/SiO_2_ substrate by mechanical exfoliation. The heights of zone 1 and zone 2 are 16.36 nm and 5.25 nm, respectively. b) ADRDM results of the same WTe_2_ flake (Figure 1a) in polar coordinates. c) Azimuth-dependent RDM images.

To characterize the optical anisotropy, a few layered 1T’-WTe_2_ flake was mechanically exfoliated and transferred onto a pre-cleaned Si/SiO_2_ wafer, and atomic force microscopy (AFM) was used to measure the selected two areas as shown in Figure 2a. Then the ADRDM measurement was conducted by rotating the incident angle (θ) of polarization from 0° to 360°, and the different values of *N*(θ) are displayed in Figure 2b as a function of incident angle. The absolute values of *N* were taken from the two selected areas of the 1T’-WTe_2_ sample, indicating a highly different reflectance with the incident angle variation. Meanwhile, Figure 2c shows the ADRDM images, which were taken at different angles with *N*(θ) changes plotted in color scale. Both the quantitative and the visualization results demonstrate that 1T’-WTe_2_ is crystalline with a highly anisotropic optical property.

### Angle-resolved DC conductance

**Figure 3:**
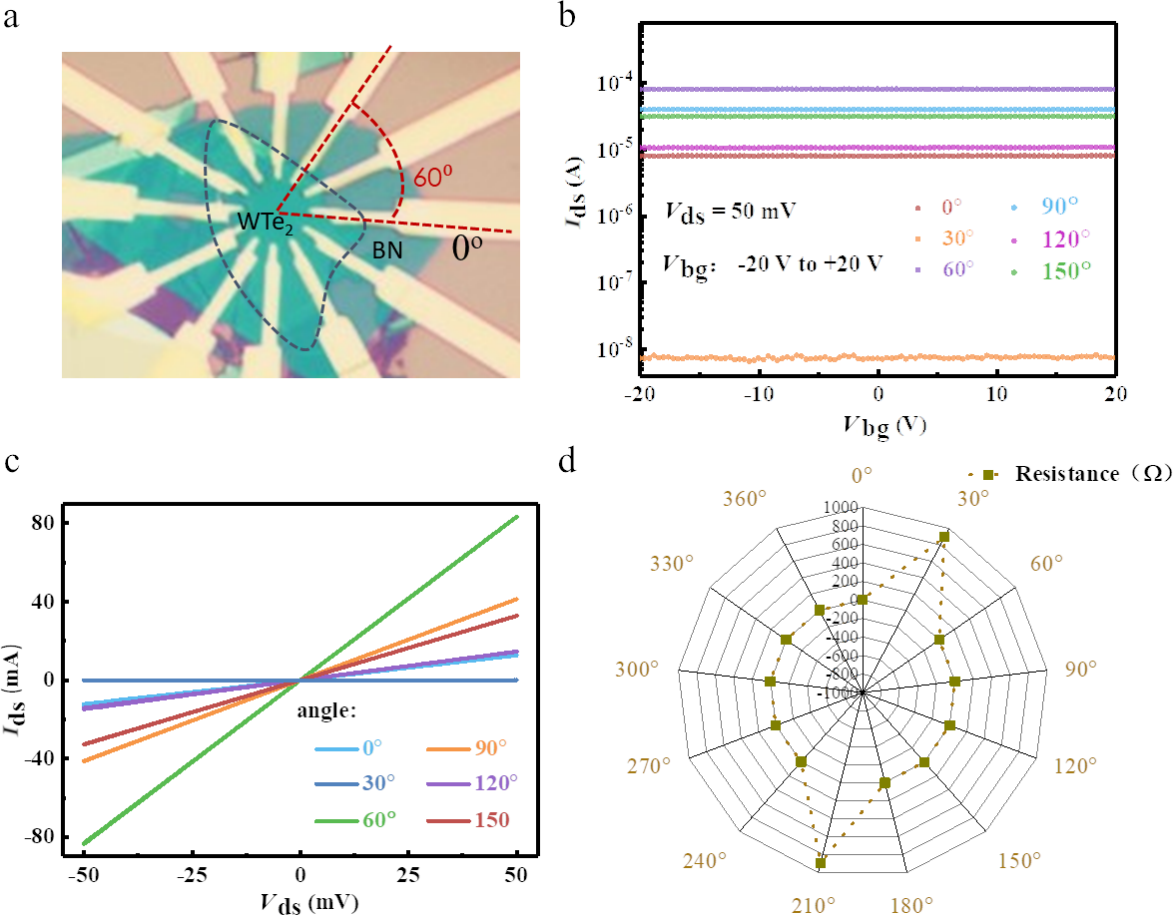
Angle-resolved DC conductivity of a 1T’-WTe_2_ thin film. a) Optical image of s WTe_2_ flake with 12 electrodes spaced 30° apart. b) and c) are the transfer characteristic curves and the output characteristic curves, respectively. d) Angle-resolved DC resistance presented as a radar map.

As mentioned above, 1T’-WTe_2_ has a distorted orthorhombic crystal structure, which is distinctly anisotropic. According to standard mobility theory, the intrinsic mobility may be influenced by anisotropic acoustic phonon scatter and elliptic elastic constants (both are relevant to the anisotropic crystalline structure) [31]. One of the consequences of an anisotropic carrier mobility is the anisotropic resistance. Therefore, we also performed angle-resolved DC conductance measurements on the 1T’-WTe_2_ flake. In detail, first we mechanically exfoliated a few-layer hexagonal boron nitride (h-BN) flake onto a cleaned Si/SiO_2_ (400 μm/285 nm) substrate with pre-fabricated metal pads (20 nm Gr/180nm Au), then the same process was conducted to transfer a 1T’-WTe_2_ flake onto a h-BN film under an optical microscope. This was followed by e-beam lithography and lift-off processing, where 12 electrodes (20 nm Gr/40 nm Au) were fabricated on the same 1T’-WTe_2_ flake spaced at an angle of 30° along a chosen reference direction (0°) as shown in Figure 3a. Two strategies were taken to ensure consistent contact resistance: 1) samples with uniform thickness were selected for device fabrication under the microscope; 2) a constant angular velocity (10 rpm) was kept when we evaporated electrodes onto the samples.

To perform the angle-resolved DC conductance measurements, a fixed electrode was chosen and defined as the reference electrode (RE), and a constant bias voltage (50 mV) was alternately applied between the other 11 test electrodes (TEs) and the reference electrode. In addition, each pair of diagonally positioned electrodes were separated by 10 μm at 180° apart and the transfer characteristic curves and the output characteristic curves were plotted in Figure 3b and Figure 3c, respectively, consist with the variation of test angles from 0° to 180°. All the obvious angle-dependent linear curves demonstrate the semi-metal phase and anisotropic electrical property of 1T’-WTe_2_. Figure 3d shows the angle-resolved resistance in radar coordinates (where θ is the angle with respect to the 0° reference electrode), which was displayed to highlight the large resistance along one direction of the sample. From the fitted data, the ratio of the maximum and the minimum resistance is extracted to be around 10^3^, indicating a highly anisotropic DC conductance of 1T’-WTe_2_, which indicates its promising application for electronic-related sensors.

### Wavelength- and polarization-resolved photoelectronic properties

**Figure 4:**
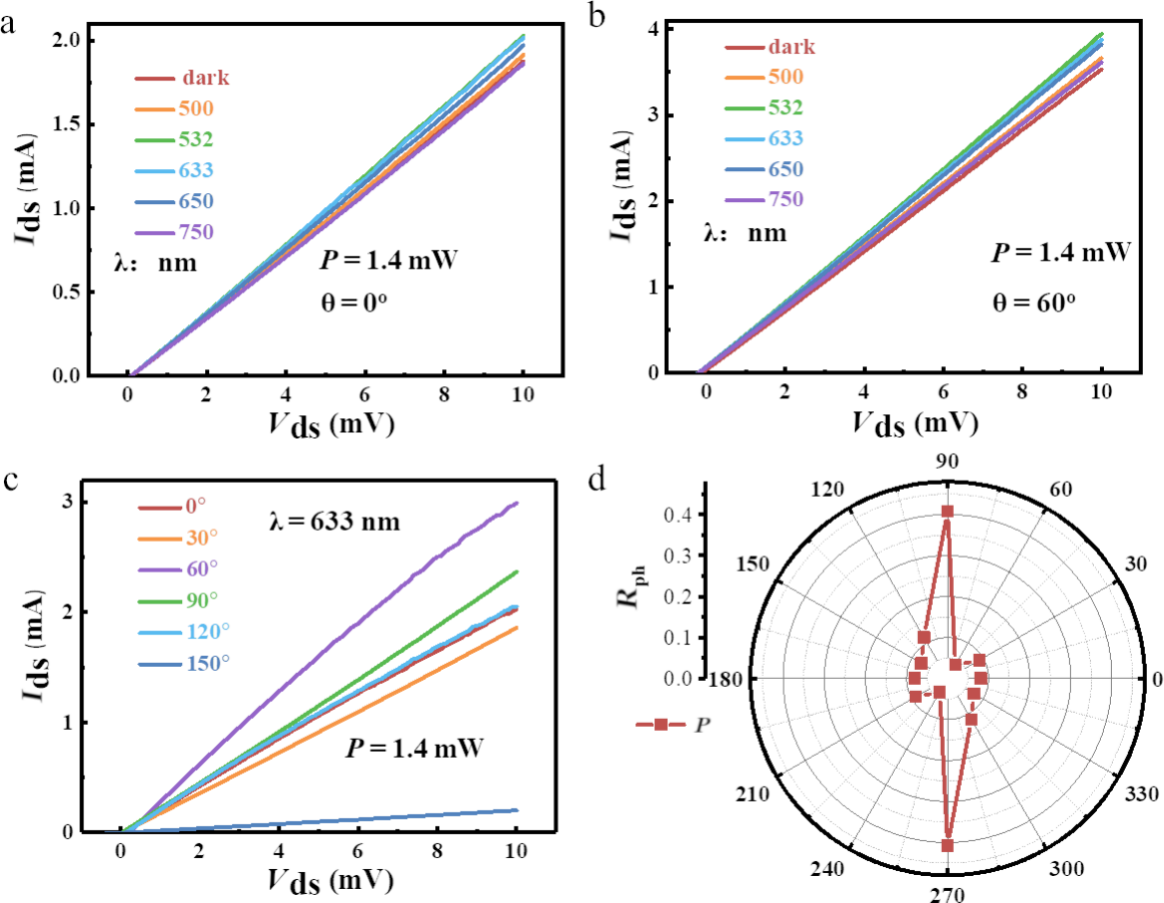
Wavelength- and angle-dependent photoelectric properties of 1T’-WTe_2_. a,b) The *I–V* curves of test angles at 0° and 60°, respectively, with incident wavelength varying from 500 to 750 nm. The incident power was set at 1.4 mW. c) *I*–*V* curves of different test angles (from 0° to 150°) with incident wavelength at 633 nm and power at 1.4 mW. d) Angle-resolved photosensitivity of 1T’-WTe_2_ in polar coordinates (wavelength: 633 nm).

The photoelectric nature of the of 1T’-WTe_2_ material was further probed by performing *I–V* measurements at room temperature in a dark environment, as presented in Figure 4a and Figure 4b. We swept the source–drain bias voltage from 0 mV to 10 mV, with a standard wavelength-tunable laser (beam diameter: 10 mm) illuminating the 1T’-WTe_2_ conductive channel. The illumination power was held at fixed power of 1.4 mW (power density: 1.78 mW/cm^2^). Then a strategy similar to the above-mentioned DC test was implemented to obtain the wavelength-dependent photocurrent. As is shown in Figure 4a and Figure 4b, in contrast to the current in dark environment (*I*_dark_), both 0° and 60° *I*_ds_ curves show a distinguishable increase in the incident wavelength variation from 500 to 750 nm, which indicates a positive, wavelength-related photoconductance of the 1T’-WTe_2_ device. On the other hand, a different response amplitude of the same illumination wavelength in terms of the different test TE/RE pairs of 0° and 60°, respectively, can be observed by comparing Figure 4a with Figure 4b. Thus, the angle-dependent photosensitivity can be observed from the two figures.

To further probe the angle-resolved photosensitivity of our device, we first held the incident laser at fixed values (wavelength: 633 nm, power 1.4 mW), then swept the source–drain voltage from 0 mV to 10 mV, and varied the angle of the TE/RE pairs from 0° to 150°. The results are presented in Figure 4c. As the test angle was changed, the *I*_ds_ increased to different degrees. Furthermore, an increase in the drain current by a factor of ≈15 was observed related to the test angles at 60° and 150°, respectively, which indicates the highly anisotropic photosensitivity of our device based on 1T’-WTe_2_. Additionally, the photosensitivity was defined as:

[3]
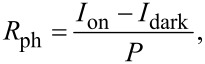


where *R*_ph_ is the photoresponsivity, *I*_on_ and *I*_dark_ are the current in the laser on and off state, respectively, and *P* is the power of the illumination laser. A more detailed observation of the polarization-resolved photosensitivity in terms of the test angle of the TE/RE pairs with variation from 0° to 360° is displayed in Figure 4d. The dramatic fluctuation of *R*_ph_ for all the test angles can be observed from the scatter symbols. The ratio of maximum/minimum value of photosensitivity reaches up to 10.7, which is attributed to the comprehensive effect of anisotropic photoelectricity and highly confined energy dispersion in 1T’-WTe_2_ [32]. Thus, the anisotropic photoresponsivity implies that 1T’-WTe_2_ has great promise for future photodetector applications.

### Angle- and wavelength-dependent photodetector

**Figure 5:**
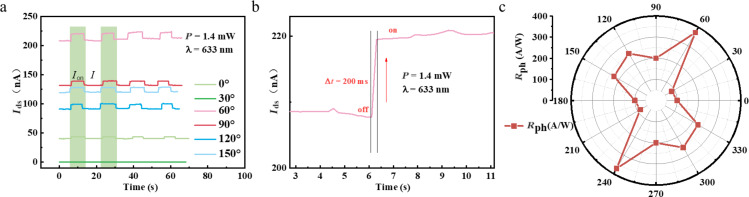
Angle-resolved photocurrent response of the photodetector based on 1T’-WTe_2_. a) Real-time photocurrent response of different test angles (from 0° to 150°, spaced at 30° apart) under the following conditions: *V*_ds_ = 5 mV, incident power = 1.4 mW, incident wavelength = 633 nm. b) The magnified photocurrent response of test angle at 60° with a response time of around 200 ms. c) Angle-resolved photosensitivity of the photodetector.

To evaluate the potential application of 1T’-WTe_2_ as a photoelectric sensor we also performed another angle-resolved photoelectric measurement. In this test, we first fixed the incident wavelength at 633 nm and the power at 1.4 mW. Then a strategy comparable with the above-mentioned electronic test was performed to get the angle-resolved photocurrent with bias voltage of 5 mV. The real-time response curves are presented in Figure 5a, in which a more distinct photoelectric effect can be observed under the daylight illumination using a household lamp. Meanwhile, a rapid increase and decrease in current reached the saturation value and the minimum value within about 200 ms, achieved by switching the laser on and off. Figure 5b shows the magnified change of the 60° photocurrent curve achieved by changing the laser state from off to on. Due to the more abundant laser-generated carriers, distinguishable fluctuations of the current values were obtained when the test angles of TE/RE pairs were varied from 0° to 150°. More detailed observations of the photosensitivity yield (*R*_ph_ = (*I*_on_ − *I*)/*P*) is presented in Figure 5c, which not only offers further evidence for highly angle-resolved photoelectricity of 1T’-WTe_2_, but also it means a potential application for an angle-dependent photodetector is promising.

**Figure 6:**
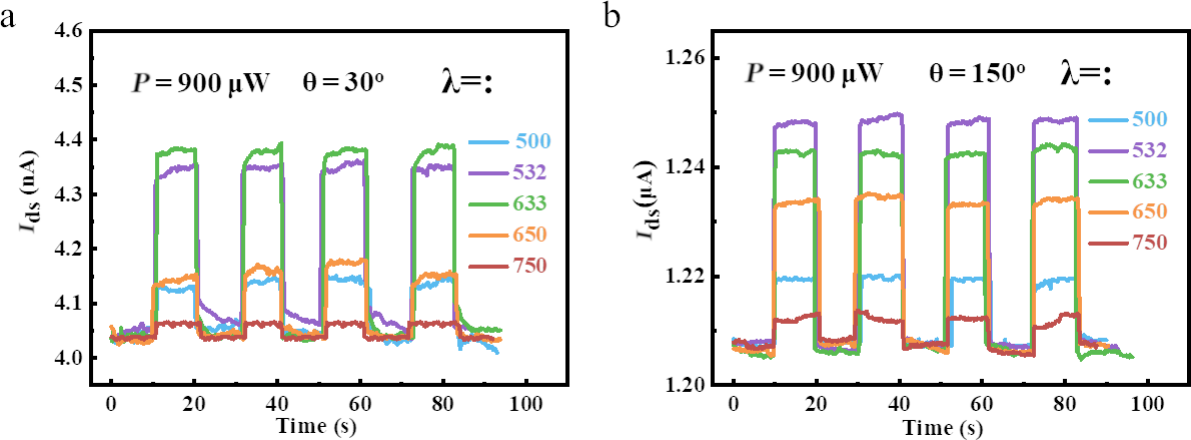
Wavelength-resolved photocurrent response of the photodetector fabricated with 1T’-WTe_2_. a,b) The real-time and photocurrent response of different test angles at 30° and 150°, with incident wavelength variation from 500 to 750 nm.

Subsequently, we further studied the wavelength-dependent photodetector fabricated using 1T’-WTe_2_. In detail, a constant power value scale was set at 900 μW, and the incident wavelength of illumination laser was varied from 500 to 750 nm. Then we carried out the measurement along the two different TE/RE pairs of 30° and 150° with bias voltage at 5 mV, and the *I*_ds_ curves are displayed in Figure 6a and 6b. As observed from the two figures, the value scales of the basic current along 30° and 150° are around 4.0 nA and 1.2 μA, respectively, due of the anisotropic absorption of light. Although the basic current induced by daylight illumination turned out to be a non-negligible, the photocurrent changed immediately when the laser state was alternately switched between on and off. Both the response and recovery time are around 200 ms. Furthermore, a distinguishable wavelength-resolved phenomenon can be observed from the two figures. Thus, the 1T’-WTe_2_ material can be used for wavelength-dependent photodetectors.

## Conclusion

In summary, with the combination of XPS spectroscopy, HR-TEM, Raman spectroscopy, ADRDM and angle-resolved electrical measurements, we successfully revealed the in-plane optical and electrical anisotropy of 2D layered 1T’-WTe_2_. Furthermore, we presented the highly anisotropic photoelectric property in detail, showing a 300-fold improvement in the photosensitivity. Moreover, due to its attractive properties, mono- or few-layer 1T’-WTe_2_ was shown to be a promising, novel and fascinating material for high-performance magnetoresistance applications in thin-film electromagnetics [33–35]. In addition, its highly anisotropic properties hold promise for a new direction in the development of angle-resolved optoelectronic and electronic devices. For instance, 1T’-WTe_2_ is a natural material for designing polarizers and polarization sensors in the broadband spectral range because of its semi-metal bandgap structure and high anisotropy. In addition to angle-dependent photodetectors, its angle-resolved photoelectric properties may permit the development of plasmonic devices in which the surface plasmon polariton frequency has a highly directional dependence on the wave vector. Besides, the semi-metal bandgap structure allows various energy band engineering methods [36–37] to be explored for the promising application in electronic and photonic devices [38–41].

In general, due to its highly anisotropic nature, 1T’-WTe_2_ offers optimistic prospects for the applications in novel device concepts by utilizing its angle-resolved and wavelength-dependent properties.

## Methods

### Exfoliation and characterization of 1T’-WTe_2_ flakes

The 1T’-WTe_2_ single crystals were purchased from XianFeng Nano Corporation. A blue film was used to exfoliate 1T’-WTe_2_ onto the N^++^-doped Si substrate covered with 285 nm of SiO_2_ for Raman spectroscopy, ADRDM and electrical characterization. The substrate had pre-patterned alignment grids and 12 electrodes (20 nm Gr/180 nm Au). XPS analysis was performed on a VG Scientific ESCALAB 250 device. The TEM images and SAED patterns were performed with on a FEI Tecnai F20 platform with an acceleration voltage of 200 kV. A HORIBA HR800 Raman spectrometer equipped with a 532 nm wavelength laser was used for the Raman spectroscopy analysis. Additionally, the thickness of 1T’-WTe_2_ was confirmed using AFM (Bruker Dimension Icon) based on the sample thickness.

### Electrical characterization

All of the electrical characterization experiments were performed using a Keysight B1500A semiconductor device analyzer and a probe station with micromanipulation probes.
