# Correction: Remarkable electronic and optical anisotropy of layered 1T’-WTe_2_ 2D materials

**DOI:** 10.3762/bjnano.10.187

**Published:** 2019-09-23

**Authors:** Qiankun Zhang, Rongjie Zhang, Jiancui Chen, Wanfu Shen, Chunhua An, Xiaodong Hu, Mingli Dong, Jing Liu, Lianqing Zhu

**Affiliations:** 1 School of Instrument Science and Opto-Electronics Engineering, Beijing Information Science and Technology University, No.12 Xiaoying East Road, Beijing, China; 2 School of Precision Instrument and Opto-Electronics Engineering, Tianjin University, 92 Weijin road, Tianjin 300072, China

**Keywords:** 1T'-WTe_2_, 2D material, anisotropic, light polarization, optoelectrical

The authors wish to add the following sentence to the Acknowledgement section:

The optical anisotropic analysis of WTe_2_ was performed on the ADRDM system developed in Prof. Chunguang Hu’s group at Tianjin University.

The new Acknowledgement section should be

